# Antitumor activity of the PI3K δ-sparing inhibitor MEN1611 in PIK3CA mutated, trastuzumab-resistant HER2 + breast cancer

**DOI:** 10.1007/s10549-023-06895-2

**Published:** 2023-03-13

**Authors:** Alessio Fiascarelli, Giuseppe Merlino, Stefania Capano, Simone Talucci, Diego Bisignano, Alessandro Bressan, Daniela Bellarosa, Corrado Carrisi, Alessandro Paoli, Mario Bigioni, Patrizia Tunici, Clelia Irrissuto, Massimiliano Salerno, Joaquin Arribas, Elisa de Stanchina, Maurizio Scaltriti, Monica Binaschi

**Affiliations:** 1 Menarini Group, Preclinical and Translational Sciences, Menarini Ricerche SpA, Via Tito Speri 10, 00071 Pomezia, Rome Italy; 2 Cancer Research Program, IMIM (Hospital del Mar Medical Research Institute), Barcelona, Spain; 3 Preclinical and Translational Research Program Vall d’Hebron Institute of Oncology (VHIO), 08035 Barcelona, Spain; 4 Centro de Investigación Biomédica en Red de Cáncer, 28029 Monforte de Lemos, Madrid Spain; 5 Department of Biochemistry and Molecular Biology, Universitat Autónoma de Barcelona, Campus de la UAB, 08193 Barcelona, Bellaterra Spain; 6 Institució Catalana de Recerca I Estudis Avançats (ICREA), 08010 Barcelona, Spain; 7 Molecular Pharmacology Program, Memorial Sloan Kettering Cancer Center, New York, NY USA; 8 Department of Pathology, Memorial Sloan Kettering Cancer Center, New York, NY USA

**Keywords:** MEN1611, PIK3CA mutations, Trastuzumab resistance, PI3K inhibitor, Patient-derived xenografts

## Abstract

**Purpose:**

Dysregulation of the PI3K pathway is one of the most common events in breast cancer. Here we investigate the activity of the PI3K inhibitor MEN1611 at both molecular and phenotypic levels by dissecting and comparing its profile and efficacy in HER2 + breast cancer models with other PI3K inhibitors.

**Methods:**

Models with different genetic backgrounds were used to investigate the pharmacological profile of MEN1611 against other PI3K inhibitors. In vitro studies evaluated cell viability, PI3K signaling, and cell death upon treatment with MEN1611. In vivo efficacy of the compound was investigated in cell line- and patient-derived xenografts models.

**Results:**

Consistent with its biochemical selectivity, MEN1611 demonstrated lower cytotoxic activity in a p110δ-driven cellular model when compared to taselisib, and higher cytotoxic activity in the p110β-driven cellular model when compared to alpelisib. Moreover, MEN1611 selectively decreased the p110α protein levels in PIK3CA mutated breast cancer cells in a concentration- and proteasome-dependent manner. In vivo, MEN1611 monotherapy showed significant and durable antitumor activity in several trastuzumab-resistant PIK3CA-mutant HER2 + PDX models. The combination of trastuzumab and MEN1611 significantly improved the efficacy compared to single agent treatment.

**Conclusions:**

The profile of MEN1611 and its antitumoral activity suggest an improved profile as compared to pan-inhibitors, which are limited by a less than ideal safety profile, and isoform selective molecules, which may potentially promote development of resistance mechanisms. The compelling antitumor activity in combination with trastuzumab in HER2 + trastuzumab-resistant, PIK3CA mutated breast cancer models is at the basis of the ongoing B-Precise clinical trial (NCT03767335).

**Supplementary Information:**

The online version contains supplementary material available at 10.1007/s10549-023-06895-2.

## Introduction

The phosphoinositide 3-kinase (PI3K) proteins are a conserved family of intracellular lipid kinases that catalyze the phosphorylation of the 3′-hydroxyl group of phosphatidylinositol and phosphoinositides [1, 2]. PI3K signaling is commonly triggered by the activation of receptor tyrosine kinases (RTKs) such as HER2 or G-protein-coupled receptors (GPCRs) with the subsequent recruitment of PI3K to the plasma membrane. After PI3Ks activation, the production of the second messenger phosphatidylinositol 3,4,5 triphosphate (PIP3) converts upstream signals into intracellular phosphorylation cascades [3, 4].

Class IA PI3Ks are heterodimers composed of a p85 regulatory subunit and one of the three p110 catalytic subunits: p110α, p110β, and p110δ (encoded by PIK3CA, PIK3CB, and PIK3CD genes, respectively). The p110α and p110β isoforms are ubiquitously expressed, while p110δ and p110γ expression is largely restricted to immune cells [5, 6]. RTK or GPCR phosphorylation triggers conformational changes in the heterodimer of PI3K, relieving the p110 catalytic subunit from the inhibition of p85 subunit bound to the phosphorylated tyrosine of the receptor. The p110 subunit, in turn, phosphorylates PIP2 to generate PIP3. The latter acts as a second messenger, recruiting AKT to the plasma membrane and activating AKT-dependent and independent downstream signaling pathways. The activation of the PI3K signaling is normally counteracted by numerous enzymes such as the phosphatase and tensin homolog (PTEN) that removes the 3′ phosphate from PIP3 and acts as a major brake in the pathway [7, 8].

Oncogenic PI3K mutations are mainly found in the p110α isoform and are frequently associated with specific amino acid residues. The most frequent mutational hotspots are predominantly harboured in the helical (E542, E545) and kinase (H1047) domains of the protein [5–8] and result in increased PI3K membrane recruitment and associated kinase activity [9].

While p110α activity is critical to sustaining tumor growth in PIK3CA-mutant cancer cells, tumors lacking PTEN function become dependent on p110β for PI3K signaling and cell proliferation [10–12]. Tumor dependency on p110β has also been proposed as one of the potential mechanisms of resistance to the α-selective PI3K inhibitor alpelisib [13].

The PI3K isoform p110δ is essential for B-cell development and function [14], and its pharmacological inhibition has been associated with immune-related dose-limiting toxicities, as reported for the taselisib phase III trial [15, 16]. Moreover, PI3K pathway alterations are also involved in trastuzumab resistance due to de novo or acquired mechanisms [17–23,24]. For this reason, targeting the PI3K pathway in trastuzumab-resistant breast tumors might re-sensitize cancer cells to anti-HER2 therapy and improve patient outcomes [25, 26].

Here we report the characterization of MEN1611 in comparison with other PI3K inhibitors in terms of PI3K isoforms inhibition profile and the investigation of its efficacy as single agent and in combination with trastuzumab in several in vivo xenograft models of HER2 + breast cancers.

## Methods

### Drugs

MEN1611 was synthesized at Menarini Ricerche (Pisa) and dissolved in a DMSO (Sigma-Aldrich C5135)/Cremophor EL (Sigma Aldrich D2650) solution (50%/50% v/v) for the in vitro assays. MEN1611 was formulated in 10% (w/v) hydroxypropyl-beta cyclodextrin (Sigma Aldrich H107) and 10% (v/v) polyethylene Glycol 400 (Merck Millipore) for the in vivo studies. Trastuzumab, 150 mg powder concentrate for solution for infusion, was purchased from Farmacia Vaticana. Alpelisib and taselisib were purchased from ChemCruz and Cayman Chemical respectively. Idelalisib was purchased from MedChem.

### Cell viability assay

To assess the effect of MEN1611 and alpelisib on cell viability, breast cancer cells were plated in 96-well tissue culture plates and treated for 72 h at 37 °C, 5% CO2. Cytotoxicity was evaluated with AlamarBlue Cell Viability Reagent (Thermo Fisher Scientific DAL1100) according to the manufacturer’s instructions, and fluorescence was measured at 590 nm by the M200 Microplate Reader (Tecan). The antiproliferative activity (%) was calculated according to the formula: T/C* 100, where T and C represent the fluorescence from cells treated and untreated, respectively. A four parameters sigmoidal concentration–response curve was then used to calculate the IC_50_ with Prism software (GraphPad).

### Capillary electrophoresis immunodetection

Cell lysates were prepared using the radioimmunoprecipitation (RIPA) buffer (Thermo Fisher Scientific 89,901) supplemented with Complete Mini protease inhibitors (Roche) and PhosSTOP phosphatase inhibitors (Roche). Proteins of interest were analyzed by JESS (Protein Simple Inc.) capillary electrophoresis-based immunodetection system. Reagents and equipment were purchased from Protein Simple and samples were analyzed following the manufacturer’s instructions. Peak area calculations were performed by the Compass software, using the Gaussian method for peak fitting. Regarding the evaluation of p110α degradation, for each sample analysed, the peak area of PI3K p110α was normalized to the peak area of the housekeeping GAPDH and then reported as a percentage of the normalized peak area of the control. All experiments were performed in triplicate and mean ± SD was showed as a bar plot. Anti-phospho-AKT S473 (#4060) and anti PI3K p110α (#4249) antibodies were purchased from Cell Signaling Technology, and the anti-GAPDH antibody was from SCBT.

### Immunohistochemistry

Tumor tissue nodules derived from the PDX153 study were formalin-fixed and paraffin-embedded before sliced into five-microns thick sections. After deparaffinization and re-hydration steps, sections were incubated with anti-Ribosomal Protein (Ser240/244) (Cell Signaling Technology #5364) or anti-Phospho-Akt (Ser473) (Cell Signaling Technology #4060) both diluted 1:50. Immunostaining was then developed by Dako REAL™EnVision™Detection System, Peroxidase/DAB + , Rabbit/Mouse (Dako K5007).

### Statistical analysis

GraphPad Prism Software (GraphPad Software Inc.) was used for statistical analysis. Statistical differences were considered to be significant at P < 0.05 using the two-tailed Mann–Whitney rank test. In vivo data are presented as mean, with the value for each group represented as a symbol of different shape and line of different color.

### Supplementary material

Detailed methods are available in Online Resource 1 for the following protocols: Immunoblotting, Isolation of healthy donors derived PBMCs, Flow cytometry-based cell death analysis on B-cells, Flow cytometry-based cell death analysis on PI3KCA mutant breast cancer cells, Cell line-derived tumor models, Patient-derived tumor models, Modified Response Evaluation Criteria in Solid Tumors (mRECIST).

## Results

### MEN1611 has a lower cytotoxic activity toward B-cells as compared to breast cancer cells

The biochemical profile of MEN1611 is unique with respect to both pan-inhibitors and isoform-selective inhibitors as it targets α-, β-, and γ isoforms and spares the δ [27, 28]. This result was also confirmed in-silico by interrogating the “Probes and Drugs” database. According to the Potency-Selectivity score (PS), PI3Kα was classified as the first target of MEN1611, followed by PI3Kγ and PI3Kβ, whereas PI3Kδ was ranked the lowest. The same in-silico evaluation was then applied to the pan-PI3K inhibitor buparlisib and to the isoform-selective PI3K inhibitors alpelisib (α-selective inhibitor), taselisib (β-sparing inhibitor) and idelalisib (δ-selective inhibitor) (Table 1). This interesting feature of MEN1611 suggests it might exhibit an improved profile as compared to pan-inhibitors, which are impaired by a less than ideal safety profile, and isoform selective molecules, which may potentially promote the development of resistance mechanisms [29].

**Table 1:** Comparison among different PI3K inhibitors based on In-silico potency-selectivity score. Data from Probes & Drugs (https://www.probes-drugs.org/home/)

	MEN1611	Taselisib	Alpelisib	Buparlisib	Idelalisib
PI3Kα	0,46	0,67	0,66	0,37	0,01
PI3Kβ	0,17	0,17	0	0,2	0,03
PI3Kδ	0,06	0,92	0,02	0,24	0,65
PI3Kγ	0,32	0,44	0,02	0,13	0,16

**Fig. 1:**
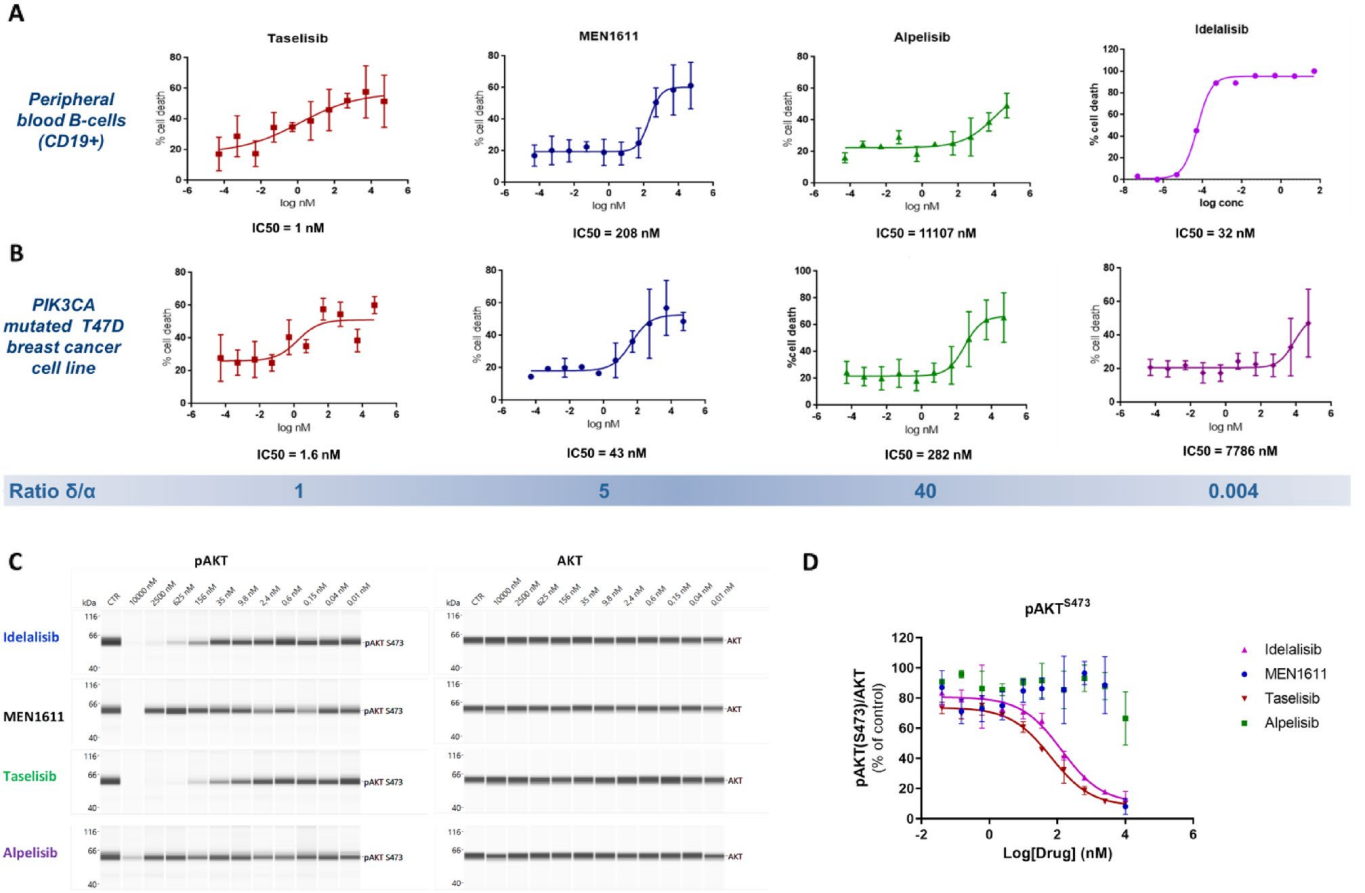
On target off-tumor activity comparison among different PI3K inhibitors: taselisib showed similar cytotoxicity on both B cells and T47D breast cancer cell (PIK3CA H1047R), while MEN1611 was significantly less toxic on B cells focusing to an intra-drug analysis. The same was for alpelisib, which showed higher activity on breast cancer cells compared to B cells. Idelalisib, as expected, demonstrated poor activity on breast cancer setting and high activity on B cells **(a, b)** Inhibition of pAKT in a PI3K delta-driven model (immortalized B-cells) after 2 h of drug exposure. Capillary immunoassay analysis of phospho and total AKT following treatment with MEN1611 and other PI3Ki. Representative lane view image of phospho an total AKT protein levels (A). Means ± SD of pAKT protein levels normalized to total AKT and expressed as percentage of DMSO-treated samples (*N* = 3) **(c, d)**

For this reason, we decided to assess the ability of MEN1611 and of competitor compounds (alpelisib, taselisib and idelalisib) to spare the PI3Kδ isoform in comparison with their ability to target the PI3Kα isoform, using PI3Kδ- and in PI3Kα-driven cellular models, respectively. As B-cells are dependent on PI3Kδ, (1–3) healthy donor PBMCs were exposed to MEN1611, taselisib, alpelisib, and idelalisib and then analysed by flow cytometry to measure cell death of blood cell subpopulations and particularly of B cells. Taselisib, with an IC_50_ of 1.26 nM, was the most cytotoxic drug among all the tested compounds, confirming its ability to targeting p110δ with high affinity (Fig. 1A). MEN1611 (IC_50_ = 208 nM) showed much weaker activity than taselisib, but stronger activity than alpelisib. Idelalisib, a δ-selective PI3K inhibitor approved for relapsed chronic lymphocytic leukemia [30]**,** was used as a positive control and showed strong activity against p110δ driven cells (IC_50_ = 32 nM). Using the same experimental approach, we examined cell death induced by these inhibitors in the PIK3CA H1047R mutated breast cancer cell line T47D (Fig. 1B), which is an ideal model for PI3Kα dependency. Taselisib displayed the strongest potency (IC_50_ = 1.59 nM), followed by MEN1611 (IC_50_ = 42.56 nM) and alpelisib (IC_50_ = 282.2 nM). As expected, idelalisib showed poor activity against this breast cancer cell line (IC_50_ = 7786 nM).

In addition, the cytotoxic effects observed in B cells with the tested inhibitors was paralleled by suppression of pathways downstream of PI3K. Indeed, in a human immortalized B-Lymphoblastoid cell line (B-LCL), taselisib strongly suppressed the phosphorylation of AKT with an IC_50_ comparable to that of idelalisib. In contrast, MEN1611 was ineffective in inhibiting AKT phosphorylation at pharmacologically relevant concentrations, similar to observations with alpelisib (Fig. 1C-D)*.*

The results indicate that, in contrast to taselisib and other pan-inhibitors, MEN1611 displays a greater potency against PI3Kα than PI3Kδ at clinically relevant concentrations, as demonstrated by a higher IC_50_ ratio between the targeting of p110α- versus p110δ-dependent models (IC_50_ normal B cells/IC_50_ T47D cancer cells of 4.89 for MEN1611 versus 0.79 for taselisib).

### MEN1611 induces a clinically relevant cytotoxic effect in breast cancer cell lines with PTEN-loss

**Fig. 2:**
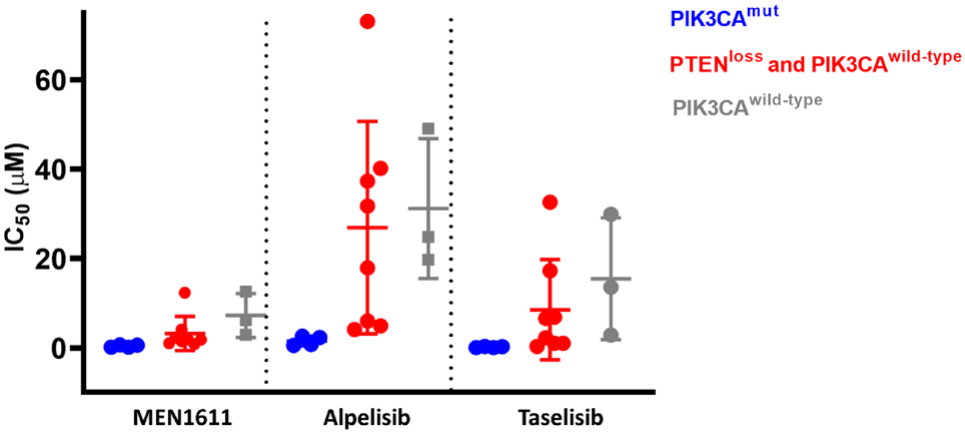
Cell viability assay in breast cancer cell lines**.** MEN1611 showed an improved in vitro antiproliferative potential in p110β-driven cellular models (PTEN loss breast cancer cells) compared to alpelisib and taselisib, while the antiproliferative activity in PIK3CA H1047R and K111N mutated breast cancer cell lines was similar among all drugs

To further explore the unique biochemical profile of MEN1611, specifically, its ability to target both alpha and beta isoforms (Table 1), we challenged its effectiveness in cell lines characterized by PIK3CA or PTEN alterations (Online Resource 2, Table 2). Breast cancer tumors carrying PTEN loss of function mutations have been shown to rely on the p110β catalytic subunit to activate the PI3K signalling pathway. MEN1611 showed stronger activity than alpelisib (from 3 to almost 22 folds) in affecting cell viability in cells characterized by PTEN-loss that are wild-type for PIK3CA (Fig. 2A). Taselisib showed an average IC_50_ in PTEN-deficient cell lines lower than alpelisib but higher than MEN1611, in line with the reduced activity of the compound against the beta isoform. In contrast, the activity of all three inhibitors on PIK3CA-mutated cells was comparable in terms of IC_50_.

### MEN1611 reduces p110α protein levels in PIK3CA mutated breast cancer cell lines

Recent studies have demonstrated that some PI3K inhibitors, such as taselisib and inavolisib, more potently inhibit mutant PI3K pathway through selective degradation of mutant p110α [31]. Based on these data, the ability of MEN1611 to induce degradation of p110α was tested.

**Fig. 3:**
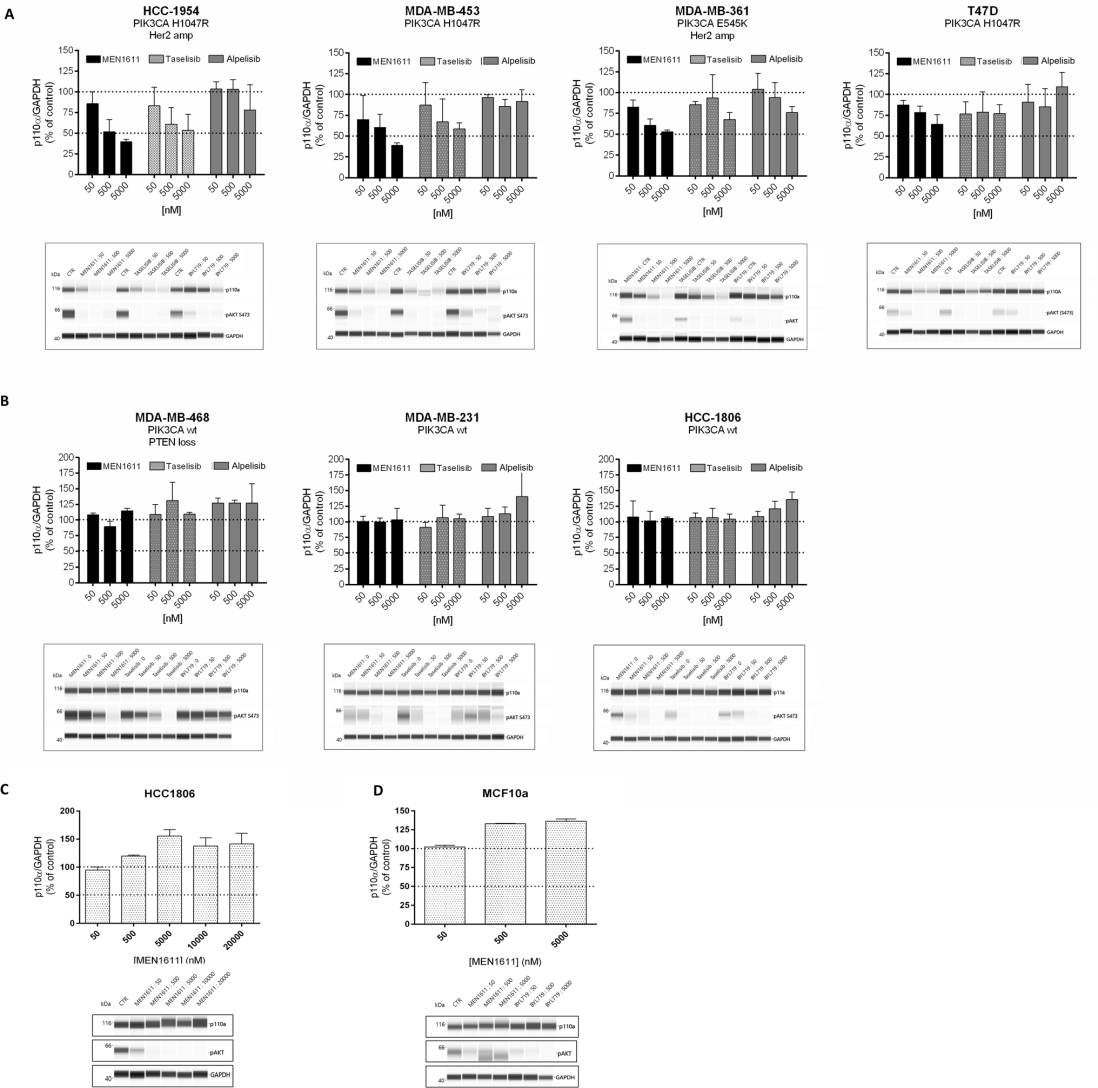
p110α protein levels in breast cancer cell lines treated with MEN1611 and other PI3K inhibitors. Analysis of p110α protein and pAKT levels in breast cancer cell lines treated with MEN1611, taselisib and alpelisib. Cell lines were treated for 24 h with the indicated concentrations and analyzed by capillary immunoblot. The levels of p110α were normalized to GAPDH and expressed as percentage of the control. The inhibition of the PI3K signaling was evaluated by determination of pAKT levels. Results are presented as mean ± SD (*N* = 3) and for each experiment a representative lane view image was showed. Effect of MEN1611, BYL719 and taselisib on p110α protein level and downstream signalling in PIK3CA mutated, and (**a**) wild-type breast cancer cell lines (**b**)**. **Effect of MEN1611 on p110α protein level in HCC1806 (PIK3CA wt) cell line treated with a wide range of concentration (**c**)**. **Effect of MEN1611 on p110α protein level in the non-tumorigenic human breast epithelial cell line MCF10A (**d**)

Several breast cancer cell lines with different PIK3CA mutation and HER2 amplification statuses were treated with MEN1611, alpelisib and taselisib. As expected, all the PI3K inhibitors suppressed the PI3K downstream signalling pathway in all the cell lines tested as showed by reduction in AKT phosphorylation. MEN1611 and taselisib also induced the degradation of the p110α protein in a dose-dependent manner but only in breast cancer cell lines harboring PIK3CA mutations (H1047R and E545K) independently of HER2 status (Fig. 3 A, B). In contrast, depletion of p110α protein levels after alpelisib treatment was negligible in the PIK3CA mutated cell lines (Fig. 3A-B). The inability of MEN1611 to induce any depletion of p110α in PIK3CA wild-type cell lines was also observed at very high MEN1611 concentrations (Fig. 3C; Online Resource 2, Table 2). No degradation was induced by MEN1611 when tested in the non-tumorigenic human breast epithelial cell line MCF10A, further supporting the ability of MEN1611 to selectively degrade the oncogenic p110 protein (Fig. 3D).

To further characterize the depletion of p110α induced by treatment with MEN1611, we evaluated whether the reduction in p110α protein levels was mediated by proteasomal degradation. Co-treatment of HCC1954 cell line with MEN1611 and the proteasome inhibitor MG132 impaired p110α depletion in comparison to samples treated only with MEN1611 (Online Resource 2, Fig. 6). Importantly, the degradation of p110α by MEN1611 was selective, as p110β protein levels were not affected by MEN1611 in any of the cell lines tested, including those characterized by PTEN loss (Online Resource 2, Fig. 7).

### MEN1611 restores trastuzumab sensitivity in HER2 amplified, PIK3CA mutated xenografts and PDX models of breast cancer

**Fig. 4:**
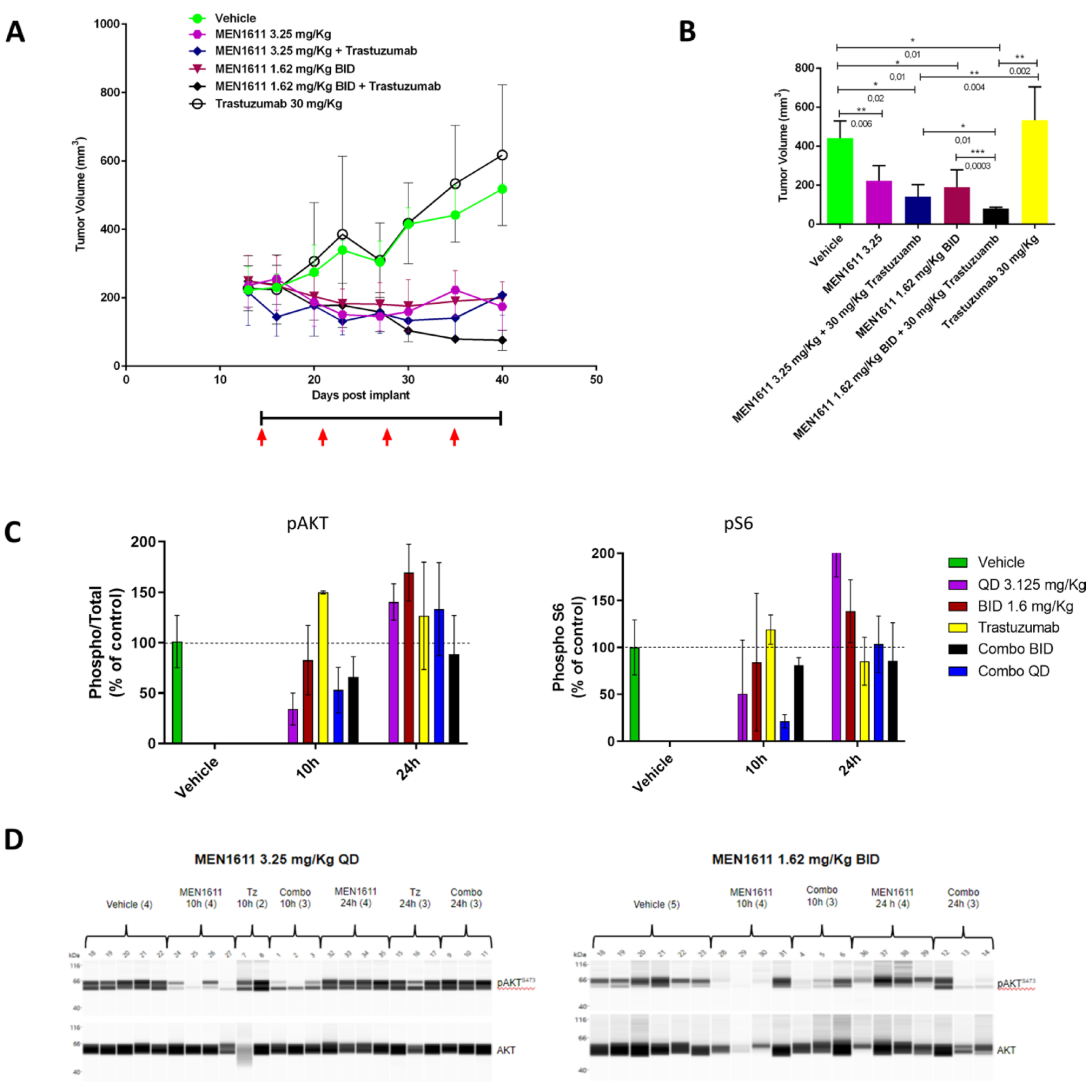
Antitumor activity and PD of MEN1611 and combination in the JIMT-1 cell-derived xenograft tumor model. Tumor cells were injected s.c. into CD-1 nude mice at day 0 and drug dosing started on day 15. Black line and red arrows represent MEN1611 and trastuzumab dosing respectively. **a** Tumor volume and statistical analysis of vehicle and treated groups according to the Mann Whitney test, evaluated at day 50. JIMT-1 cell-derived xenograft breast cancer model was treated with MEN1611 given p.o. once-daily (QD) at 3.25 mg/Kg versus twice-daily (BID) at 1.6 mg/Kg. At the indicated time points the group of mice (3 mice/group) were sacrificed and tissues were collected. Each tumor mass was dissociated in RIPA buffer and analyzed by capillary immunoassay for pAKT S473, AKT, pS6 S240-244 and S6. **b** The level of phospho-protein were normalized to total protein and expressed as percentage of the control **(c)** Lane view for pAKT and total AKT **(d)**

To further investigate the antitumor activity of MEN1611, we used xenograft models refractory to trastuzumab due to HER2-independent activation of the PI3K pathway [40]. Two cell line-derived xenograft models, JIMT-1 and HCC1954, harboring C420R and H1047R mutations in the PIK3CA gene, respectively, were initially used to evaluate the activity of MEN1611 and trastuzumab given alone or in combination, with a concomitant administration schedule. In both models, the combination showed greater antitumor activity than either drug given in monotherapy (Fig. 4A and Online Resource 2, Fig. 8 and 9).

In particular, in the JIMT-1 xenograft model, we investigated the activity of MEN1611 in combination with trastuzumab in two different administration schedules: once-daily (QD) and twice-daily (BID).

MEN1611 as a single agent demonstrated significant tumor volume inhibition in QD (3.25 mg/kg) as well as in BID (1.6 mg/kg × 2) schedules. The antitumor activity was paralleled by PI3K pathway inhibition as evaluated by pAKT inhibition.

A significant improvement in tumor regression in the BID (82% TVI) in comparison to QD group (68% TVI) was achieved when MEN1611 was combined with trastuzumab (Fig. 4A-B). The signaling was suppressed continuously when the BID schedule was assessed at a clinically achievable dose, and partially counteracted the rebound effect observed at the 24 h time point in the QD group (Fig. 4C-D).

**Fig. 5:**
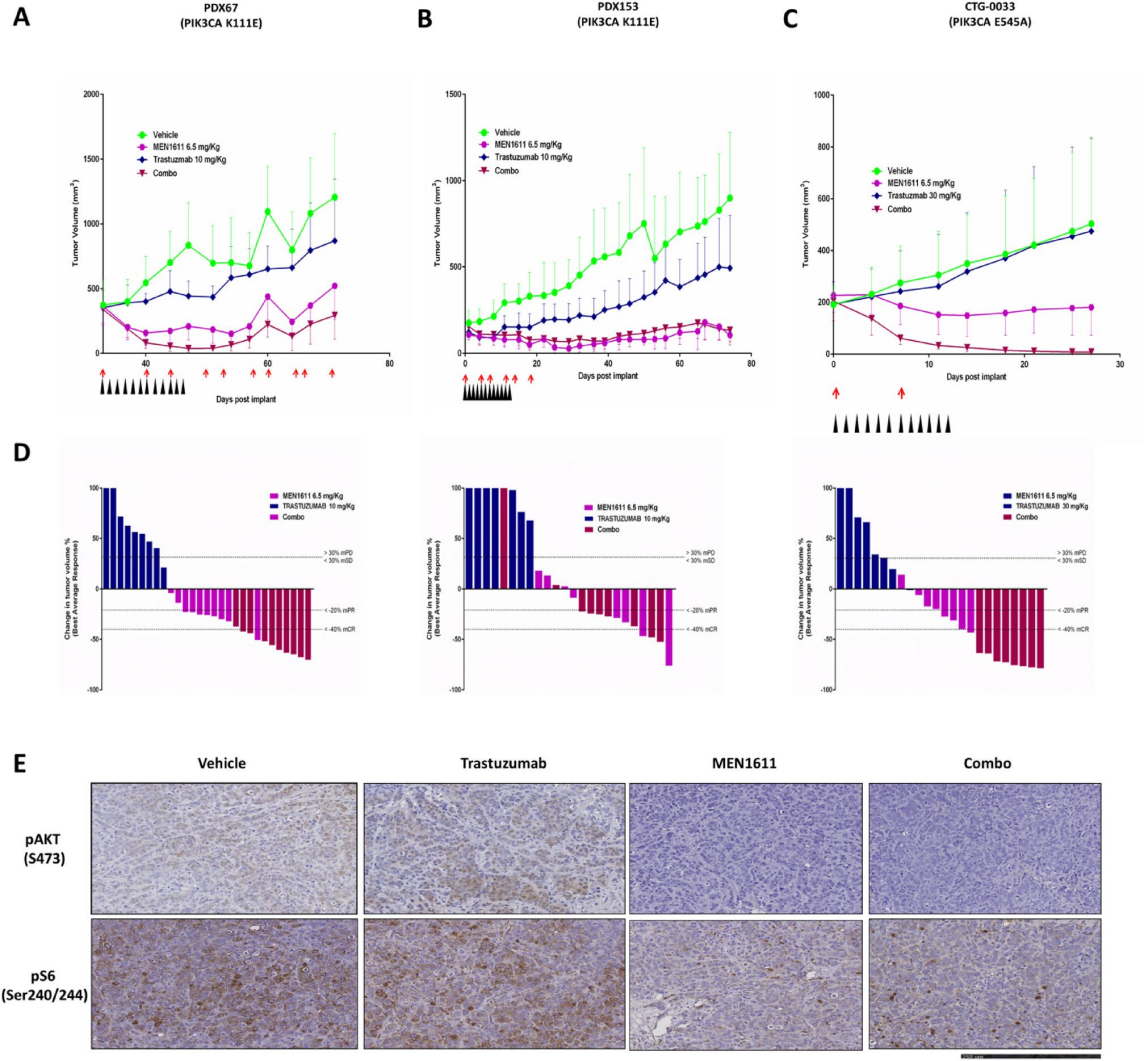
MEN1611 antitumor efficacy in patient-derived xenograft models of breast cancer harboring PIK3CA mutations and resistance to trastuzumab. PDX67, PIK3CA K111E (**a**) PDX153, PIK3CA K111E (**b**) CTG-0033, PIK3CA E545A (**c**) Waterfall plots in response to treatment were calculated according to the mRECIST criteria at the end of the study, with each bar represent a single mouse. mCR, complete response; mPR, partial response; mSD, stable disease; mPD, progressive disease. Black and red arrows represent MEN1611 and trastuzumab dosing respectively (**d**) Representative immunostaining for phosphorylated AKT (pAKT) and phosphorylated S6 (pS6) in PDX153 by immunohistochemical analysis (**e**)

To confirm and strengthen the results observed with cell-line xenografts, we treated three patient-derived xenografts (refractory to trastuzumab and carrying PIK3CA mutations) with MEN1611, trastuzumab and the combination. In PDX67 and CTG-0033 MEN1611 showed the ability to restore trastuzumab sensitivity, resulting in a stronger anti-tumor response in the combination cohorts compared to single agents. In PDX153 both the single agent and the combination treatment resulted in a stronger anti-tumor response compared to Trastuzumab treatment (Fig. 5).

In the PDX153 and CTG-0033 PDX models, we observed a striking and durable activity of the combination treatment with 100% of the animals cured in the PDX CTG-0033 model (Fig. 5C). Treatment was well tolerated, and no adverse toxicities were observed. The relevance of the tolerability profile of MEN1611 in mouse is supported by the very high degree of sequence homology between the mouse and human PI3K isoforms.

To evaluate in vivo target engagement and support the strong antitumor efficacy observed in the combination group, tumor masses from PDX153 were harvested at the end of treatment and analyzed by IHC for phospho-protein levels. IHC analysis of PDX153 tumour nodules revealed that the combination therapy induced a complete inhibition of AKT phosphorylation and a dramatic inhibition of pS6 (Fig. 5D). These results confirm the strong suppression of PI3K signalling and support the long-lasting antitumor efficacy achieved by the combination of MEN1611 and trastuzumab.

## Discussion

In this paper, we report the striking and durable activity of MEN1611 in combination with trastuzumab in HER2 positive-breast cancer models characterized by PI3Kα mutations.

PI3Ks represent a central hub that regulates many key processes of the cell such as cell growth, survival, proliferation and differentiation, key hallmarks in the development of cancer [3]. Enormous drug discovery efforts have been made to develop a pharmacological strategy to inhibit this attractive target [32]. Many PI3K inhibitors have indeed been tested in clinical trials but have shown poor efficacy and tolerability [33, 34] until alpelisib was approved in combination with fulvestrant in 2019, demonstrating that the PI3K-targeting strategy can be clinically feasible and meaningful [35].

Nevertheless, identifying the optimal PI3K isoform inhibitory profile and the most effective combination strategy in different diseases remains an important challenge.

The four different catalytic isoforms of class I PI3Ks (p110α, p110β, p110δ, and p110γ) are known to play different roles in signal transduction depending on tumor histotypes and genetic background. p110α is essential for tumor driven by PIK3CA mutations and is also activated in tumors harboring alterations in RTKs and RAS; p110β seems to be the main isoform involved in PI3K signaling in tumor presenting PTEN deficiencies; and p110δ, a key PI3K isoform for B-cells development, is crucial for lymphomas [29]. Targeting solid tumors with an inhibitor with activity toward p110δ might lead to immunological side-effects [36–38].

Pan-PI3K inhibitors target all catalytic isoforms of class I PI3K and thus have the potential for broad activity across different tumor types and molecular alterations. However, it is now recognized that a broad inhibition profile could be a source of dose-limiting toxicities leading to poor efficacy. In contrast, isoform-selective PI3K inhibitors may have a wider therapeutic window, allowing the administration of the full therapeutic dose. However, this improved selectivity may lead to the fast emergence of resistance [39].

MEN1611 is a α, β, ɣ.-selective and δ- sparing PI3K inhibitor, which differentiates it from both the pan- inhibitors as well as from isoform-selective molecules.

In this report, we have highlighted the PI3K delta-sparing profile of MEN1611. Results from in vitro binding affinity and analysis of in silico data, which predict a potency-selectivity score based on on-target and off-target affinities, indicate a unique kinase inhibition profile for MEN1611 with respect to alpelisib, a p110α selective inhibitor, and taselisib, a p110β-sparing PI3K inhibitor that strongly inhibits both p110α and p110δ isoforms.

The profile of MEN1611 was also confirmed in a cell-based model known to be dependent on PI3Kδ for proliferation and survival. In healthy-donor derived B lymphocytes, MEN1611 showed a greater than 160-fold reduction in cytotoxic activity in comparison to taselisib.

Moreover, taselisib was equally cytotoxic toward both PIK3CA mutant breast cancer and B cells, targeting both with an IC50 around 1 nM. On the contrary, MEN1611 showed higher cytotoxic activity toward α-driven cancer cells compared to δ-driven healthy-donor derived B cells. The sparing of the PI3K delta isoform by MEN1611 may potentially reduce the on-target off-tumor side effects, thus differentiating MEN1611 from taselisib and from pan-inhibitors that have shown dose-limiting toxicities impairing drug efficacy.

Alpelisib targets the p110α isoform at nanomolar potency and is more than 50-fold more selective for p110α over the p110δ and p110γ isoforms and more than 200-fold more selective for p110α over the p110β isoform. However, the strong selective pressure exerted by an isoform-specific PI3K inhibitor might influence the genetic dependency of cancer cells, allowing the escape through other PI3K isoforms, such as p110β, thus leading to drug resistance, as recently demonstrated [13]. In this context, an inhibitor that targets both the p110α and p110β isoforms could have an advantage in overcoming acquired resistance mechanisms, compared to an α-selective PI3K inhibitor.

MEN1611, similar to alpelisib, targets with the highest potency the p110α wild-type and mutated isoforms, but is more effective than alpelisib in inhibiting the growth of cancer cell lines with PIK3CA-wt and PTEN-loss genetic background. The improved efficacy of MEN1611 over alpelisib in these cells could be the result of the dual inhibition of p110α and β by MEN1611.

Recently it has been reported that the some PI3K inhibitors, such as taselisib and inavolisib, have the ability to behave as monomeric degrader and can selectively induce the degradation of the p110α mutated protein in addition to their ability to inhibit p110α catalytic activity [31]**.**

In this study, we show that MEN1611, as well as taselisib but not alpelisib, is able to induce a dose-dependent decrease in p110α protein levels. The MEN1611-mediated p110α depletion occurs selectively in PIK3CA mutant breast cancer cell lines at a concentration range in which cytotoxic effects are observed. As MEN1611 degrades mutant PI3Kα but does not affect *α* and *β* wild-type protein levels, a more durable effect of MEN1611 in PI3Kα-mutated cancers might be possible. Thus, MEN1611 unique biochemical profile translates into a peculiar and promising pharmacological profile.

We next focused our efforts in investigating the efficacy of MEN1611 in combination with trastuzumab in clinically relevant preclinical models representing HER2 + breast cancer.

Among HER2-positive breast cancers, approximately 20% harbor a PIK3CA mutation that is associated with resistance to trastuzumab-based therapy [17, 40]. Indeed, PI3K oncogenic mutations can activate the PI3K signaling pathway in a HER2-independent manner, rendering HER2 inhibition ineffective. MEN1611 monotherapy showed antitumor activity in several HER2-positive trastuzumab-resistant PIK3CA-mutant PDXs harboring different concomitant genomic aberrations, subsequently inducing tumor-stasis or tumor regression. The combination of MEN1611 with trastuzumab significantly improved antitumor efficacy compared to single agent treatment, overcoming trastuzumab resistance and resulting in a long-lasting response.

In summary, we have used both in vitro and in vivo breast cancer models to characterize the profile of MEN1611 in comparison to pan-inhibitor and isoform-selective inhibitors. The pharmacological profile of MEN1611 suggests that there may be potential implications on drug tolerability and ability to overcome resistance. Moreover, MEN1611 in combination with trastuzumab showed a significant and long-lasting antitumor response in HER2-positive trastuzumab-resistant models. Overall, these results support the B-Precise clinical study (Clin Trial.gov NCT03767335), a Phase Ib trial of MEN1611 in combination with trastuzumab ± fulvestrant for the treatment of HER2-positive advanced or metastatic breast cancer.

## Supplementary Information

Below is the link to the electronic supplementary material.

Supplementary file1 (DOCX 25 KB)

Supplementary file2 (DOCX 1083 KB)

## Data Availability

The datasets generated during and/or analysed during the current study are available from the corresponding author on reasonable request.
