# Supplementary material for: Antitumor activity of the PI3K δ-sparing inhibitor MEN1611 in PIK3CA mutated, trastuzumab-resistant HER2 + breast cancer
Source: Breast Cancer Res Treat. 2023 Mar 13;199(1):13–23. doi: 10.1007/s10549-023-06895-2 (PMC10147754; doi:10.1007/s10549-023-06895-2)
Supplement: Supplementary file 1 — Supplementary file1 (DOCX 25 KB) [file 10549_2023_6895_MOESM1_ESM.docx]

Antitumor activity of the PI3K δ-sparing inhibitor MEN1611 in PIK3CA mutated, trastuzumab-resistant HER2+ breast cancer

Breast Cancer Research and Treatment

Alessio Fiascarelli^1^, Giuseppe Merlino^1^, Stefania Capano^1^, Simone Talucci^1^, Diego Bisignano^1^, Alessandro Bressan^1^, Daniela Bellarosa^1^, Corrado Carrisi^2^, Alessandro Paoli^1^, Mario Bigioni^1^, Patrizia Tunici^1^, Clelia Irrissuto^1^, Massimiliano Salerno^2^, Joaquin Arribas^3-4-5-6-7^, Elisa de Stanchina^8^, Maurizio Scaltriti^9^, Monica Binaschi^1^

^1^Menarini Group, Preclinical and Translational Sciences, Pomezia, Rome, Italy ; ^2^Menarini Group Preclinical and Translational Sciences, Pomezia, Rome, Italy at the time the data were generated; ^3^Cancer Research Program, IMIM (Hospital del Mar Medical Research Institute), Barcelona, Spain; ^4^Preclinical and Translational  Research Program Vall d’Hebron Institute of Oncology (VHIO), Barcelona, 08035, Spain; ^5^Centro de Investigación Biomédica en Red de Cáncer, Monforte de Lemos, Madrid, 28029, Spain; ^6^Department of Biochemistry and Molecular Biology, Universitat Autónoma de Barcelona, Campus de la UAB, 08193, Bellaterra, Spain; ^7^Institució Catalana de Recerca i Estudis Avançats (ICREA), 08010, Barcelona, Spain; ^8^Molecular Pharmacology Program, Memorial Sloan Kettering Cancer Center, New York, NY, USA; ^9^Department of Pathology, Memorial Sloan Kettering Cancer Center, New York, NY, USA at the time data were generated.

Alessio Fiascarelli

Menarini Ricerche SpA, Via Tito Speri 10, 00071, Pomezia, Rome, Italy

Email: [afiascarelli@menarini-ricerche.it](mailto:afiascarelli@menarini-ricerche.it)

Phone numbers: 00390691184465

Fax: 3906-9100-220

**Online Resource 1: Supplemental Methods**

**Immunoblotting**

To assess the effect of MEN1611 on the PI3K/AKT signaling pathway, JIMT-1 and HCC1954 xenograft tumor nodules were flash-frozen in liquid nitrogen and then homogenized with the BD Medimachine System in ice-cold RIPA buffer supplemented with protease and phosphatase inhibitors. Tumor lysates were centrifuged and supernatants stored at -80°C until used. Protein concentration was measured by the Pierce Pierce BCA Protein Assay Kit (Thermo Fisher Scientific 23225). Thirty-five µg of total proteins were resuspended in Laemli buffer, loaded onto 4-15% Criterion TGX stain-free SDS-gels (Bio-Rad), and electrophoresed. Proteins were then transferred onto 0.2 µm polyvinylidene fluoride (PVDF) membranes (Bio-Rad). After incubation in blocking solution (TBS-Tween and 5% BSA), membranes were probed overnight at 4°C with the following antibodies purchased from Cell Signaling Technology: rabbit anti-human Akt (#9272), rabbit anti-human phospho-Akt Ser473 (#4060), rabbit anti-human S6 ribosomal protein (#2217), and rabbit anti-human phosphor-S6 ribosomal protein (#5364). After washing with TBS-Tween, membranes were incubated for 1 hour with the appropriate HRP-conjugated secondary antibody. Signals were then detected by enhanced chemiluminescence (ECL) substrate Super Signal West Pico (Thermo Fisher) and visualized with the digital imaging system UVItec Cambridge. Densitometric quantification of the electrophoretic bands was performed using the UVItec Alliance softwar

**Isolation of healthy donors derived PBMCs**

Healthy donor-derived human buffy coats were purchased from Biopredic International (France). Human buffy coats were maintained at room temperature until processed and then diluted 1:2 with PBS 5% BSA. Next, 36 ml of buffy coats diluted with PBS containing 5% BSA were carefully layered over 16 ml of Lymphoprep (StemCell Technologies) in a 50 ml conical tube. Samples were centrifuged at 400 x g for 30 min at 20°C in a swinging-bucket rotor without brake. The mononuclear cell layer was aspirated and carefully transferred in a new 50 ml conical tube. The conical tube was then filled with Medium (RPMI 1640 containing 10% FBS, 2nM L-Glutamine, and 50 U/ml PenStrep), mixed, and centrifuged at 300 x g for 10 min at 20°C. The supernatant was then completely removed, and the tube was filled with the medium at a concentration of 2 x 10^6^ cells/ml. IL-2 (R&D Systems) was added to the PBMC culture at a final concentration of 10 ng/ml.

**Flow cytometry-based cell death analysis on B-cells**

To assess the effect of MEN1611, taselisib, alpelisib, and idelalisib on cell death of B-cells, PBMCs (1x10^6^) were incubated in the presence of the PI3K inhibitors (concentration ranging from 0.00005 to 50.000 nM) for 48 hours. At the end of the incubation, cells were washed, resuspended in PBS containing 5% of BSA, and then incubated with the mouse anti-human CD19 APC (BD) in the dark for 20 min at 4°C. After two washing steps, cells were mixed with the Annexin V-PE plus 7AAD solution (7AAD/AnnexinV kit BD), and samples were examined with the FACS Fortessa X20 using the FCS Express 6 software (Flow Research Edition). The evaluation of apoptotic/death cells was obtained by gating the CD19 positive B cells and focusing on the double-stained 7AAD/AnnexinV cell population. A four parameter sigmoidal concentration-response curve was then used to calculate the IC_50_ with Prism software (GraphPad).

**Cell line-derived tumor models**

JIMT-1 cells (DSMZ; cell line number ACC 589) are T2N1M0 stage, grade 3, ductal breast cancer cells that carry an amplified HER-2 oncogene, a p.C420R mutation in PI3KCA, and is insensitive to HER-2-inhibiting drugs. Cells were cultured in DMEM medium (Gibco) supplemented with 10% Fetal Bovine Serum (Gibco), 1% Penicillin/Streptomycin (Gibco), 1% Glutamine (Gibco). Cells were incubated at 37°C in a 5% CO2 humidified atmosphere.

HCC1954 cells, obtained from the American Type Culture Collection (ATCC), are derived from a stage IIA, grade 3, ductal breast cancer tumor carrying an amplified HER2 gene. These cells were cultured in RPMI 1640 medium (Gibco) supplemented with 10% Fetal Bovine Serum (Gibco), 1% Penicillin/Streptomycin (Gibco) and 1% Glutamine (Gibco). Cells were incubated at 37°C in a 5% CO2 humidified.

For the breast cancer xenograft model, 20 x 10^6^ JIMT-1 and HCC1954 cells were re-suspended in 0.2 mL of basement membrane extract type 3 (Trevigen) at 5.6 mg/mL and then injected subcutaneously into the right flank of 6-8 week old athymic nude mice (CD-1 nu/nu, Charles River, Calco, Italy).

Mice were maintained in microisolator cages under continuously monitored environmental conditions. Drinking water and specific sterilized diet (VRF1, Charles River) were supplied ad libitum. Environmental conditions, as well as the procedures for housing and handling the animals, were in compliance with the UKCCCR guideline (4) and the European Convention for the protection of vertebrate animals used for experimental and other scientific purposes (2010/63/EU; ref 5).

Twice a week, tumor growth and body weight were evaluated and recorded. Dimension of the tumors was measured by a caliper, and tumor masses were calculated using the following formula: length (mm) × width2 (mm) × d/2, assuming density, d = 1 mg/mm3 for tumor tissue (6).

When average tumor volume reached 200-300 mm3, animals were randomly assigned into groups of 6-7 mice/group. Group I received vehicle only. Group II received 6.5 mg/kg MEN1611 by oral gavage for 12 consecutive days (q1dx12). Group III received 30 mg/kg trastuzumab by i.p. once weekly for 2 consecutive weeks (q7dx2) and Group IV received MEN1611 and trastuzumab in combination.

Treatment effectiveness was assessed as Tumor Volume Inhibition % (TVI%) in treated versus control mice, using the following formula: (1- tumor volume of treated mice / tumor volume of control mice)*100. Mice were sacrificed when the tumors reached a volume around 10% of total body weight or when mice body weight decreased by more than 20% compared to control animals over a period of 7 days or more. Animals were euthanized with carbon dioxide exposure according to the standard procedure

**Flow cytometry-based cell death analysis on PI3KCA mutant breast cancer cells**

T47D (ATCC) breast cancer cell line was cultured in RPMI 1640 supplemented with 10% FBS and L-Glutamine. For the cell death assay, T47D cells were plated in a 6-well plate (5x10^5^/well) and cultured for 48 hours with a concentration range of 0.00005 – 50 nM of MEN1611, taselisib, alpelisib, or idelalisib. At the end of the incubation, the medium was removed, and the attached cells were left in the dissociation buffer TripLE Express Enzyme (1X) phenol red (Life Technologies) to facilitate their detachment from the plate. Cells were then harvested, washed twice with PBS 5% BSA and then resuspended in 100 µl PBS /5%BSA before the staining. To each tube, 5 µl of 7AAD and 5 µl of Annexin V-PE were added before incubating for 15 min at RT in the dark. After the incubation, 400 µl of buffer solution 10X, diluted 1:10 with sterile distilled water (7AAD/Annexin V kit BD cat. 559763), were added to each tube. Samples were then analyzed by FACS Fortessa X20 and the data were processed using the FSC Express 6. software.

**Patient-derived tumor models**

The CTG-0033 model was performed at Champions Oncology. Briefly, immunocompromised female nude mice, from Harlan Laboratories (Indianapolis, IN, USA) between 5-8 weeks of age were housed on irradiated, Alpha-twist-enriched 1/8” corncob bedding (Sheperd) in individual HEPA ventilated cages (Innocage® IVC, Innovive USA) on a 12-hour light-dark cycle at 68-74°F (20-23°C) and 30-70% humidity. Animals were fed with water (reverse osmosis, 2 ppm Cl2) and an irradiated test rodent diet (Teklad 2919; 19% protein, 9% fat, and 4% fiber) ad libitum. Mice were implanted with tumor fragments from Champions TumorGraft® model CTG-0033. When the tumor volume (TV) reached approximately 150-300 mm3, animals were randomized into groups and treatments were initiated. Group I received vehicle only. Group II received 6.5 mg/kg MEN1611 by oral gavage for 12 consecutive days (q1dx12). Group III received 30 mg/kg trastuzumab by i.p. once weekly for 2 consecutive weeks (q7dx2) and Group IV received MEN1611 and trastuzumab in combination. Tumor growth was monitored twice a week using digital calipers and the TV was calculated using the formula (0.52*[length*width^2^]). Body weight was determined twice a week using a digital scale. The studies were terminated when the mean tumor volume in the control group reached approximately 1500 mm3 or up to Day 60, whichever occurred first. Treatment effectiveness was determined by assessing Tumor Volume Inhibition % (TVI%) in treated versus control mice, using the following formula: (1- tumor volume of treated mice / tumor volume of control mice)*100.

The PDX67 model was performed at Vall d’Hebron Institute of Oncology. Briefly, 6 week-old female NOD/SCID mice (NOD.CB17/AlhnRj-Prkdc Scid, Janvier Labs) were orthotopically implanted with breast tumor fragments into the mammary fad pad or subcutaneously implanted with breast cancer tumor pieces of approximately 3x3mm embedded in Matrigel. Breast PDX models were maintained with 17-ß-estradiol (1μM) in the drinking water throughout the study. PDX tumors were measured with calipers twice a week. Once tumors reached a volume of 150-200 mm3, mice were randomized and treated. MEN1611 and trastuzumab were administered at 6.5 mg/kg PO once daily (QD) for 12 consecutive days (q1dx12), and 10 mg/kg IP bi-weekly for 5 consecutive weeks respectively. Mice were maintained and treated in accordance with institutional guidelines of Vall d’Hebron University Hospital Care and Use Committee. Treatment effectiveness was determined by assessing Tumor Volume Inhibition % (TVI%) in treated versus control mice, using the following formula: (1- tumor volume of treated mice / tumor volume of control mice)*100.

The PDX153 model was performed at Memorial Sloan Kettering. Briefly, the PDX was established by implanting breast tumor biopsy tissue subcutaneously in athymic nu/nu mice (Harlan Laboratories, Indianapolis IN, USA). Once grown, the tumor was extracted for genomic characterization (MSK-IMPACT) and expansion in mice. For tumor growth experiments, once the tumor volumes reached 100-200 mm^3^, animals were randomized into four treatment groups (6-8 mice per group Tumor growth and body weight were evaluated and recorded twice a week. Tumors were measured by caliper, and tumor volumes were calculated using the formula (0.52 [length x width^2^]). MEN1611 was administered at 6.5 mg/kg PO once daily (QD) for 12 consecutive days (q1dx12), and trastuzumab was administered at 10 mg/kg IP bi-weekly for 3 consecutive weeks. Treatment effectiveness was determined by assessing Tumor Volume Inhibition % (TVI%) in treated versus control mice, using the following formula: (1- tumor volume of treated mice / tumor volume of control mice)*100.

**Modified Response Evaluation Criteria in Solid Tumors (mRECIST)**

Best response to therapy was also determined by comparing tumor volume change at time t versus baseline using the following formula: % tumor volume change = ΔVol_t_ = [100x(Vol_t_ – Vol_i_) / Vol_i_] for t = one week after the last treatment. The criteria for the murine response (mRECIST) were adapted from the RECIST criteria and are defined as follows: mCR (Complete Response), Best Response < -95% and Best Average Response < -40%; mPR (Partial Response), Best Response < -50% and Best Average Response < -20%; mSD (Stable Disease), Best Response <35% and Best Average Response < 30%; mPD (Progressive Disease), not otherwise categorized**.**
