# Supplementary material for: Antitumor activity of the PI3K δ-sparing inhibitor MEN1611 in PIK3CA mutated, trastuzumab-resistant HER2 + breast cancer
Source: Breast Cancer Res Treat. 2023 Mar 13;199(1):13–23. doi: 10.1007/s10549-023-06895-2 (PMC10147754; doi:10.1007/s10549-023-06895-2)
Supplement: Supplementary file 2 — Supplementary file2 (DOCX 1083 KB) [file 10549_2023_6895_MOESM2_ESM.docx]

Antitumor activity of the PI3K δ-sparing inhibitor MEN1611 in PIK3CA mutated, trastuzumab-resistant HER2+ breast cancer

Breast Cancer Research and Treatment

Alessio Fiascarelli^1^, Giuseppe Merlino^1^, Stefania Capano^1^, Simone Talucci^1^, Diego Bisignano^1^, Alessandro Bressan^1^, Daniela Bellarosa^1^, Corrado Carrisi^2^, Alessandro Paoli^1^, Mario Bigioni^1^, Patrizia Tunici^1^, Clelia Irrissuto^1^, Massimiliano Salerno^2^, Joaquin Arribas^3-4-5-6-7^, Elisa de Stanchina^8^, Maurizio Scaltriti^9^, Monica Binaschi^1^

^1^Menarini Group, Preclinical and Translational Sciences, Pomezia, Rome, Italy ; ^2^Menarini Group Preclinical and Translational Sciences, Pomezia, Rome, Italy at the time the data were generated; ^3^Cancer Research Program, IMIM (Hospital del Mar Medical Research Institute), Barcelona, Spain; ^4^Preclinical and Translational  Research Program Vall d’Hebron Institute of Oncology (VHIO), Barcelona, 08035, Spain; ^5^Centro de Investigación Biomédica en Red de Cáncer, Monforte de Lemos, Madrid, 28029, Spain; ^6^Department of Biochemistry and Molecular Biology, Universitat Autónoma de Barcelona, Campus de la UAB, 08193, Bellaterra, Spain; ^7^Institució Catalana de Recerca i Estudis Avançats (ICREA), 08010, Barcelona, Spain; ^8^Molecular Pharmacology Program, Memorial Sloan Kettering Cancer Center, New York, NY, USA; ^9^Department of Pathology, Memorial Sloan Kettering Cancer Center, New York, NY, USA at the time data were generated.

Alessio Fiascarelli

Menarini Ricerche SpA, Via Tito Speri 10, 00071, Pomezia, Rome, Italy

Email: [afiascarelli@menarini-ricerche.it](mailto:afiascarelli@menarini-ricerche.it)

Phone numbers: 00390691184465

Fax: 3906-9100-220

**Table 2**

**
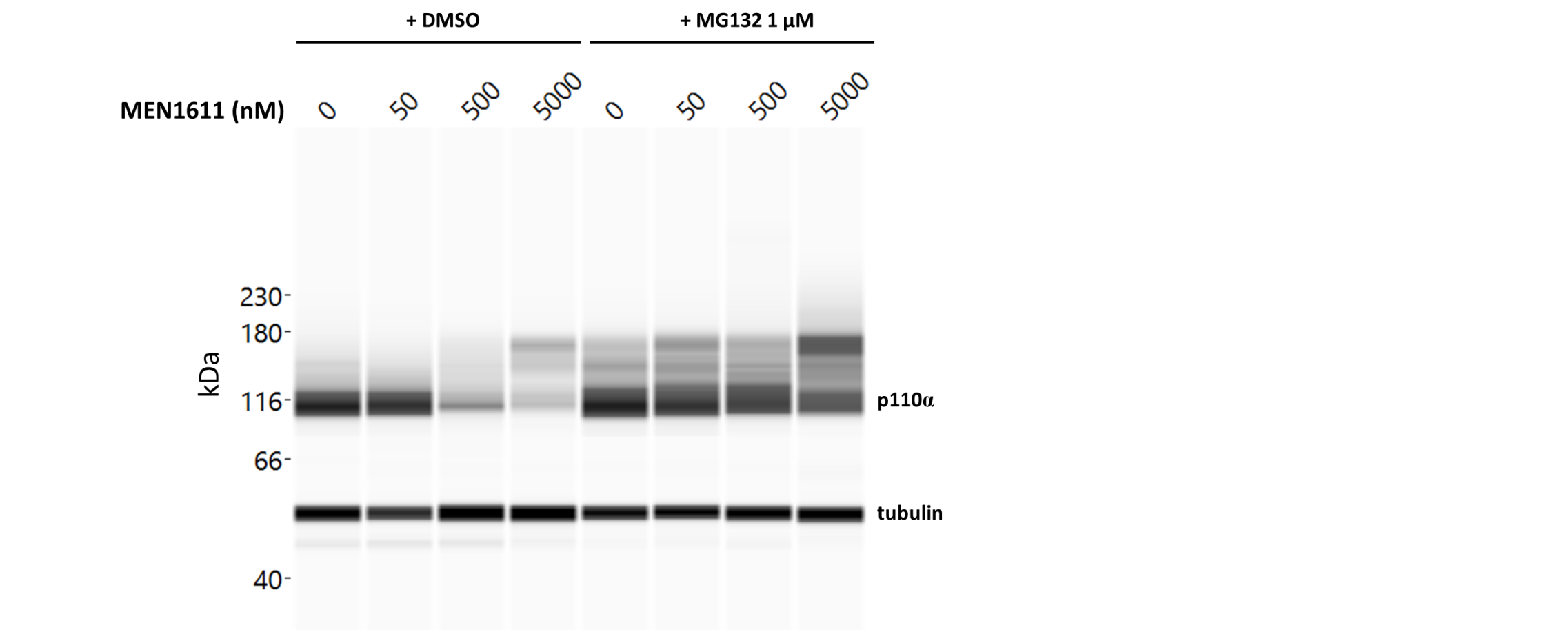
**

**Fig. 6**

**
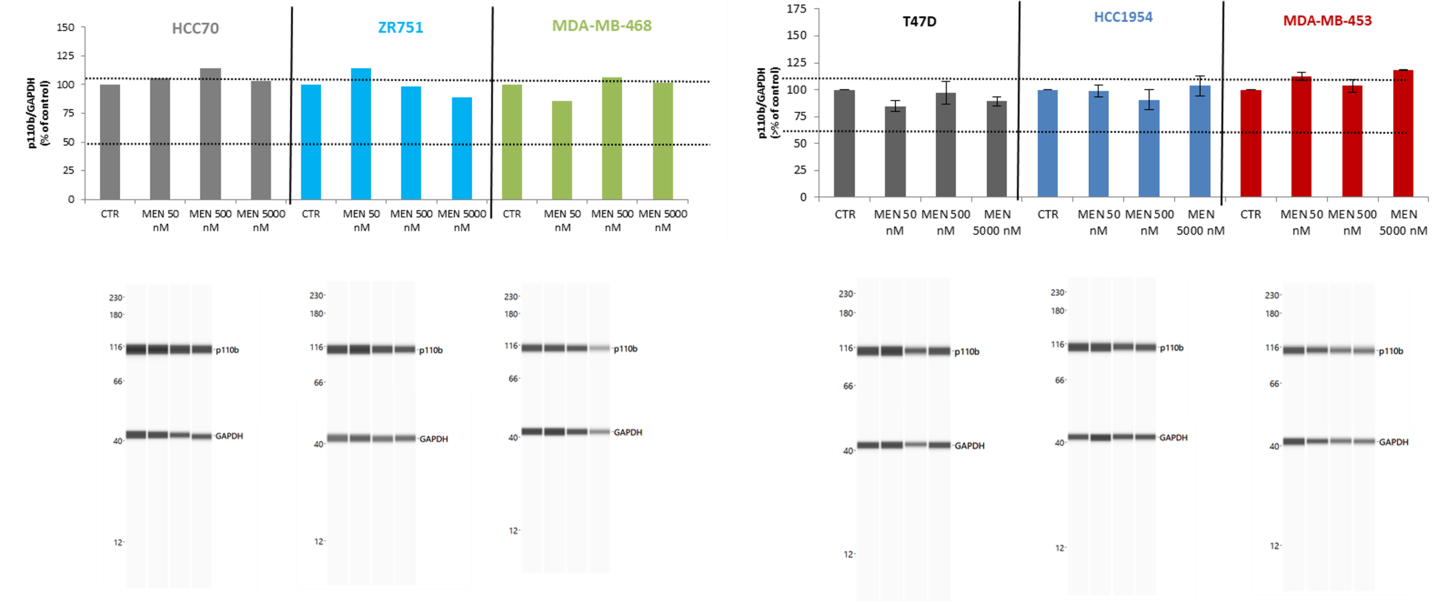
**

**Fig.7**

**
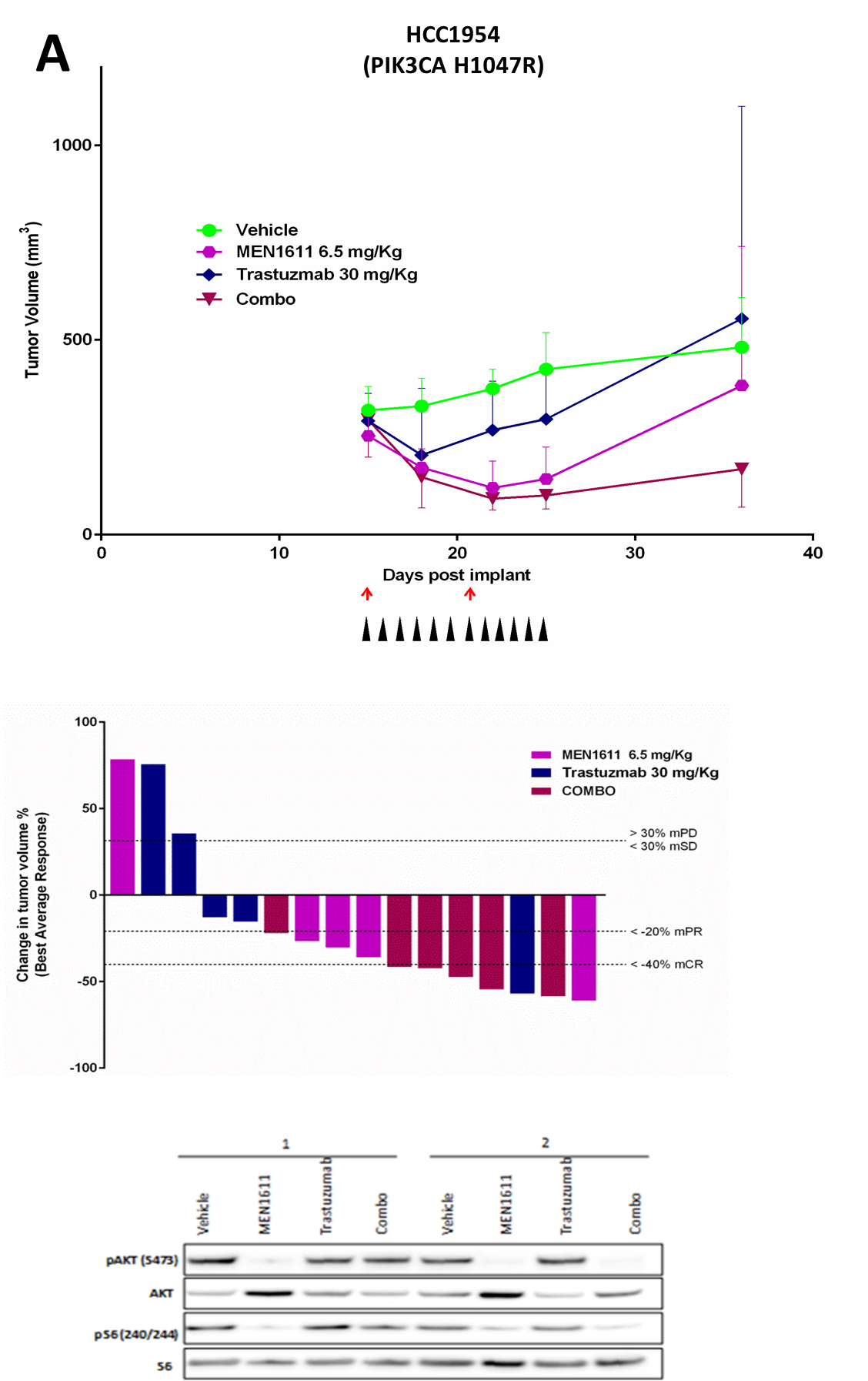
**

**Fig. 8**

**
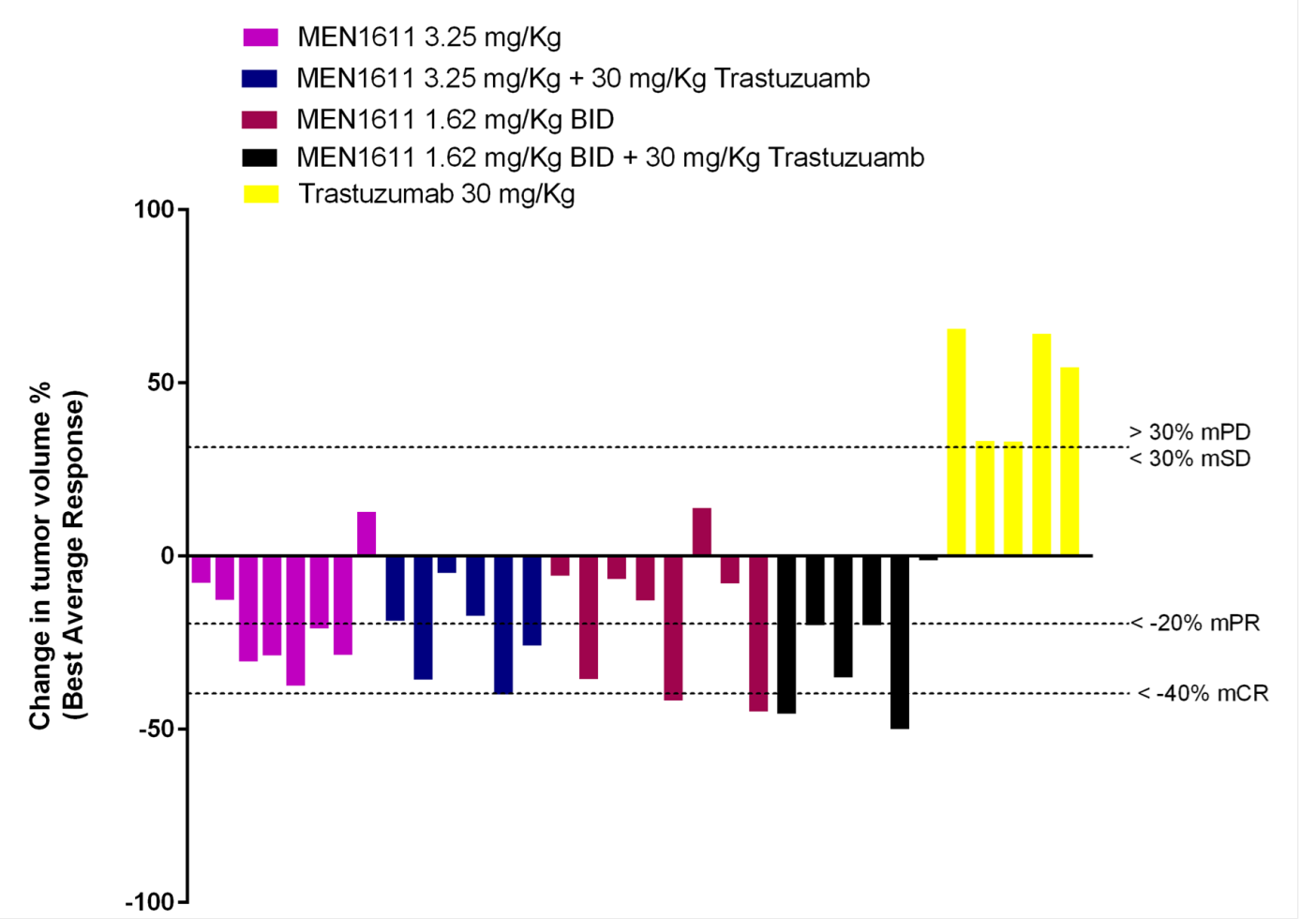
**

**Fig. 9**
